# Supplementary material for: Versatile NTP recognition and domain fusions expand the functional repertoire of the ParB-CTPase fold beyond chromosome segregation
Source: Proc Natl Acad Sci U S A. 2025 Dec 4;122(49):e2527592122. doi: 10.1073/pnas.2527592122 (PMC12704722; doi:10.1073/pnas.2527592122)
Supplement: Supplementary file 1 — Appendix 01 (PDF) [file pnas.2527592122.sapp.pdf]

Supplementary Information for

Versatile NTP recognition and domain fusions expand the functional repertoire of the ParB-CTPase fold beyond chromosome segregation

Jovana Kaljević[1†], Kirill V. Sukhoverkov[1*], Katie Johnson[1], Antoine Hocher[2†], Tung B. K. Le[1†]*

[1] Department of Molecular Microbiology, John Innes Centre, Norwich, NR4 7UH, United Kingdom

[2] Department of Genetics, University of Cambridge, Cambridge, CB2 3EH, United Kingdom

* co-first authors

† co-corresponding authors

Tung B. K. Le: tung.le@jic.ac.uk

Antoine Hocher: ah2368@cam.ac.uk

Kaljević Jovana: jovana.kaljevic@jic.ac.uk

The PDF file includes:

Figs. S1 to S9

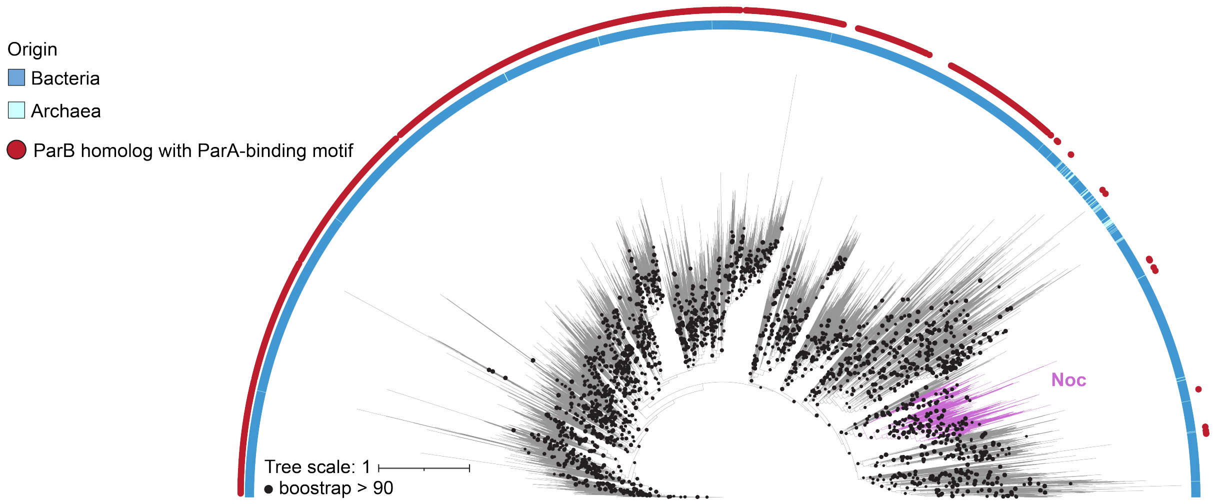

Fig. S1. Distribution of ParB proteins across bacteria and archaea. **Related to Fig. 2-3**. Midpoint rooted single gene phylogenetic tree of bacterial and archaeal ParB homologs (TIGR00180, see Methods). The inner ring indicates the taxonomic origin: blue represents bacteria, and cyan represents archaea. The outer red ring marks ParB homologues with the ParA-binding motif. Black dots denote nodes with ultrafast bootstrap support ≥ 90. The Noc protein, an example of an orphan ParB homolog that lost the ParA-binding motif, is indicated in magenta.

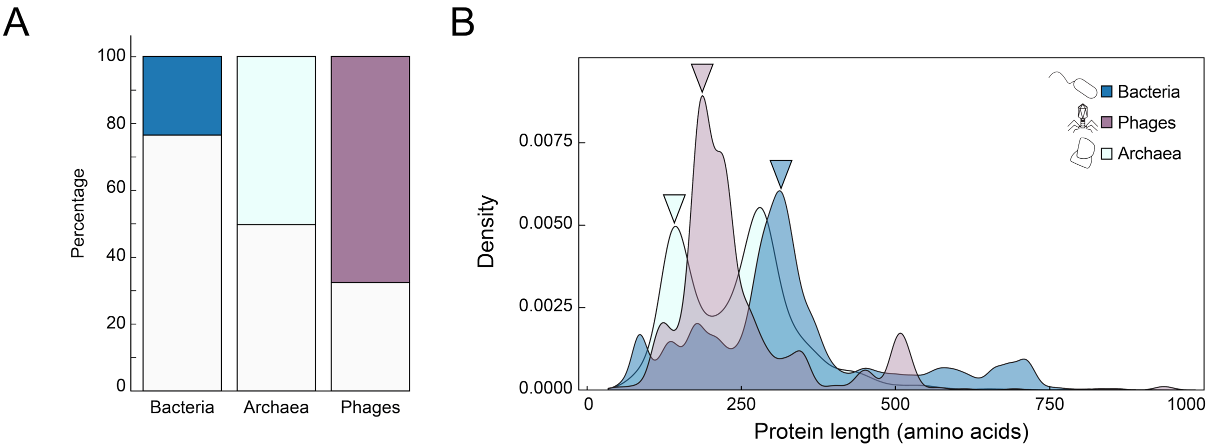

Fig. S2. Length distribution of proteins that are annotated as single-domain ParB-CTPase fold-containing homologs. Related to Fig. 4. *(A)* Fraction of ParB-CTPase fold-containing proteins annotated as "single-domain" (colored bars) versus multi-domain (grey bars, as shown in Fig. 4a) in bacteria, archaea, and phages. *(B)* Protein length distribution of the "single-domain" proteins subset, shown as density plots for bacteria (blue), archaea (cyan), and phages (purple). Short proteins (~100–200 amino acids) dominate in phages and some archaea (magenta and cyan arrowheads), consistent with a single ParB-CTPase fold. In contrast, many bacterial proteins exceed 300 amino acids (blue arrowhead), which is longer than the ParB-CTPase fold itself, suggesting that they likely contain additional domains that were not annotated.

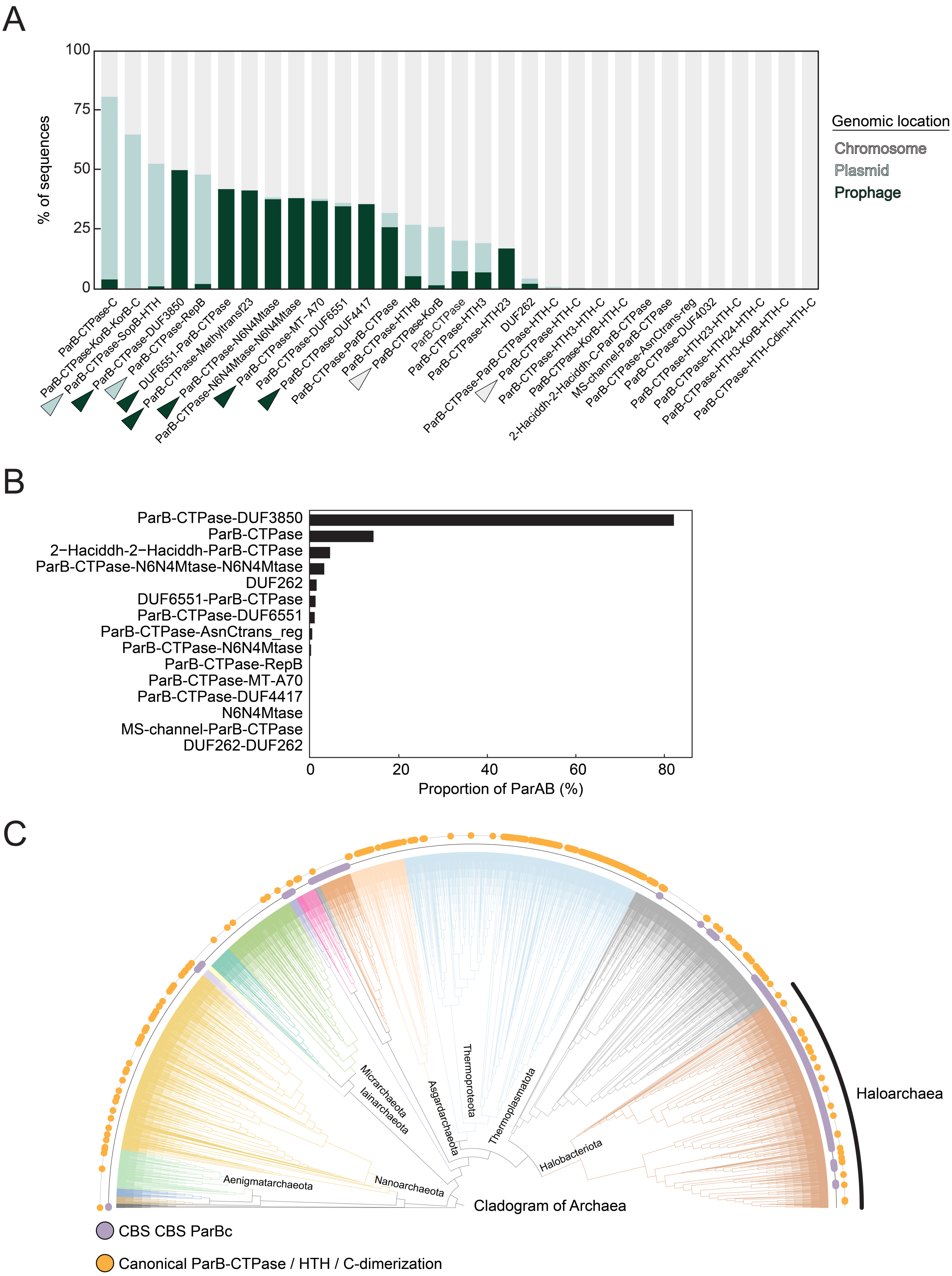

A
100
75
50
25
0
% of sequences
Genomic location
Chromosome
Plasmid
Prophage
ParB-CTPase-C
ParB-CTPase-KorB-KorB-C
ParB-CTPase-SopB-HTH
ParB-CTPase-DUF3850
ParB-CTPase-RepB
DUF6551-ParB-CTPase
ParB-CTPase-Methyltransf23
ParB-CTPase-N6N4Mtase
ParB-CTPase-N6N4Mtase-N6N4Mtase
ParB-CTPase-MT-A70
ParB-CTPase-DUF6551
ParB-CTPase-DUF4417
ParB-CTPase-ParB-CTPase
ParB-CTPase-HTH8
ParB-CTPase-KorB
ParB-CTPase
ParB-CTPase-HTH3
ParB-CTPase-HTH23
DUF262
ParB-CTPase-ParB-CTPase-HTH-C
ParB-CTPase-HTH-C
ParB-CTPase-HTH3-HTH-C
ParB-CTPase-KorB-HTH-C
2-Haciddh-2-Haciddh-C-ParB-CTPase
MS-channel-ParB-CTPase
ParB-CTPase-AsnCtrans-reg
ParB-CTPase-DUF4032
ParB-CTPase-HTH23-HTH-C
ParB-CTPase-HTH24-HTH-C
ParB-CTPase-HTH3-KorB-HTH-C
ParB-CTPase-HTH-Cdim-HTH-C
B
ParB-CTPase-DUF3850
ParB-CTPase
2−Haciddh-2−Haciddh-ParB-CTPase
ParB-CTPase-N6N4Mtase-N6N4Mtase
DUF262
DUF6551-ParB-CTPase
ParB-CTPase-DUF6551
ParB-CTPase-AsnCtrans_reg
ParB-CTPase-N6N4Mtase
ParB-CTPase-RepB
ParB-CTPase-MT-A70
ParB-CTPase-DUF4417
N6N4Mtase
MS-channel-ParB-CTPase
DUF262-DUF262
0
20
40
60
80
Proportion of ParAB (%)
C
Micrarchaeota
Iainarchaeota
Asgardarchaeota
Thermoproteota
Thermoplasmatota
Halobacteriota
Haloarchaea
Aenigmatarchaeota
Nanoarchaeota
Cladogram of Archaea
CBS CBS ParBc
Canonical ParB-CTPase / HTH / C-dimerization

Fig. S3. Distribution and genomic context of genes encoding novel ParB-CTPase fold-containing proteins. Related to Fig. 4. *(A)* Genomic location of ParB-CTPase fold-containing proteins. The x-axis lists ParB-CTPase fold-containing proteins fused to different domains. The y-axis shows the percentage of sequences for each fusion type encoded on chromosomes (grey), plasmids (blue), or prophages (dark green). Examples highlighted in text are marked with arrowheads. *(B)* Association of ParB-CTPase fold-containing proteins with the *parA* gene. Bars show the percentage of proteins that are encoded adjacent to a *parA* homolog for each domain fusion. ParB-CTPase-DUF3850 proteins are most frequently found in operons with *parA*. The DUF262 domain contains the ParB-CTPase fold. Canonical ParB proteins involved in chromosome segregation are excluded to highlight the distribution of ParB-CTPase domain fusions across other domain combinations. *(C)* Cladogram of archaeal species showing species encoding ParB-CTPase fold fused with CBS-domain (purple dots in the inner ring) and encoding ParB-CTPase fold fused with canonical DNA binding motif HTH and C-terminal dimerisation domains (orange dots in outer ring). CBS fusions are confined to specific clades, whereas canonical ParB proteins occur more patchily and typically are not encoded with a *parA* gene.

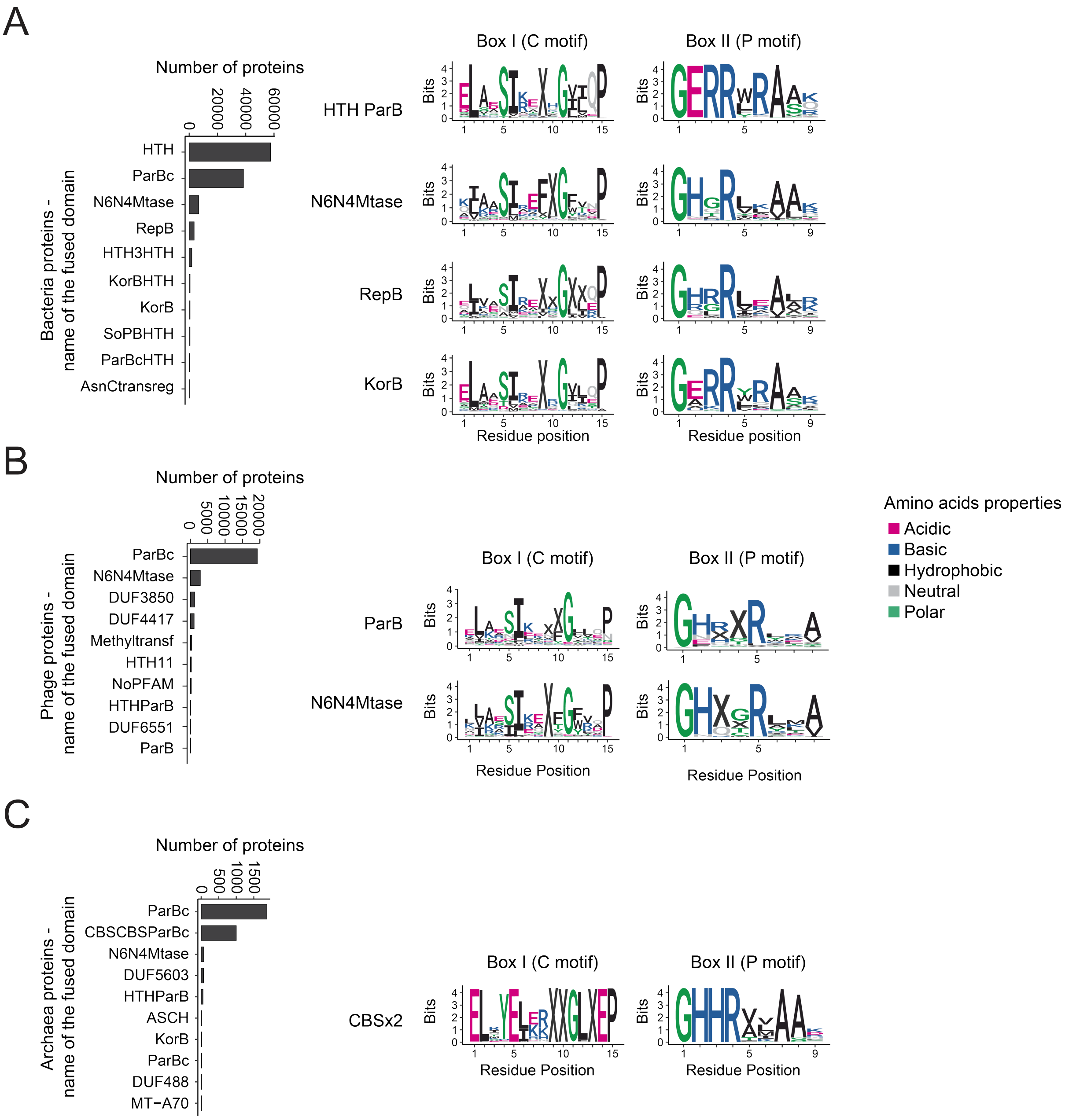

Fig. S4. Subtle variation of ParB-CTPase motifs across different ParB-CTPase fold-containing proteins. Related to Fig. 4. *(A)* Bacterial proteins. Left: frequency of domains fused to the ParB-CTPase fold. Each bar represents the number of proteins carrying the indicated fusion domain (higher bars = more frequent fusion). Right: sequence logos showing conserved Box I (C motif) and Box II (P motif) motifs for selected, most common families (e.g., HTH, N6N4-methyltransferase, RepB, KorB). *(B)* Phage proteins. Left: most frequent ParB-CTPase fusions in phages. Right: Box I (C motif) and Box II (P motif) logos for common families such as stand-alone ParB-CTPase fold proteins and N6N4-methyltransferases. *(C)* Archaeal proteins. Left: most frequent ParB-CTPase fusions in archaea. Right: Box I (C motif) and Box II (P motif) logos for the most common CBS domain fusion. Amino acids are colored based on their chemical properties.

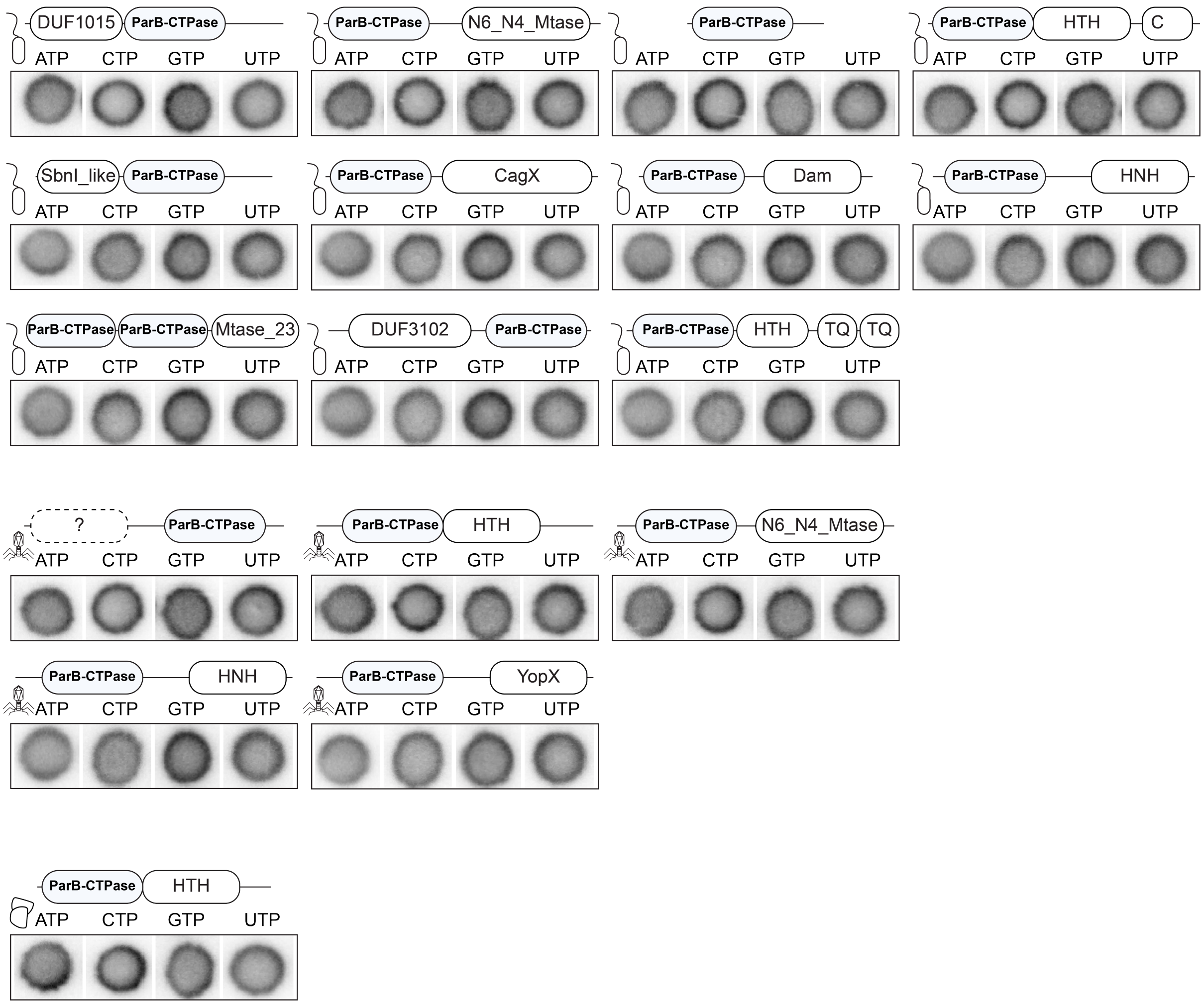

Fig. S5. Screening ParB-CTPase fold-containing proteins for nucleotide binding using DRaCALA. Related to Fig. 5. Different ParB-CTPase fold-containing proteins were tested for binding to radiolabeled NTP α-P^{32}, and none showed NTP-binding. The starting concentration of proteins used was 25 µM. DRaCALA assays were performed with two replicates; one representative replicate is shown. Cartoon icons indicate the origin of each protein (phage, bacterium, or archaea). Domain schematics above each protein indicate representative fusions, with the ParB-CTPase domain shown in blue and fused to various domains. Proteins are grouped by their origin. Detailed information for each protein (including source organism, accession numbers, and domain annotations) is provided in Dataset S5. See also *SI Appendix,* Fig. S9*D*.

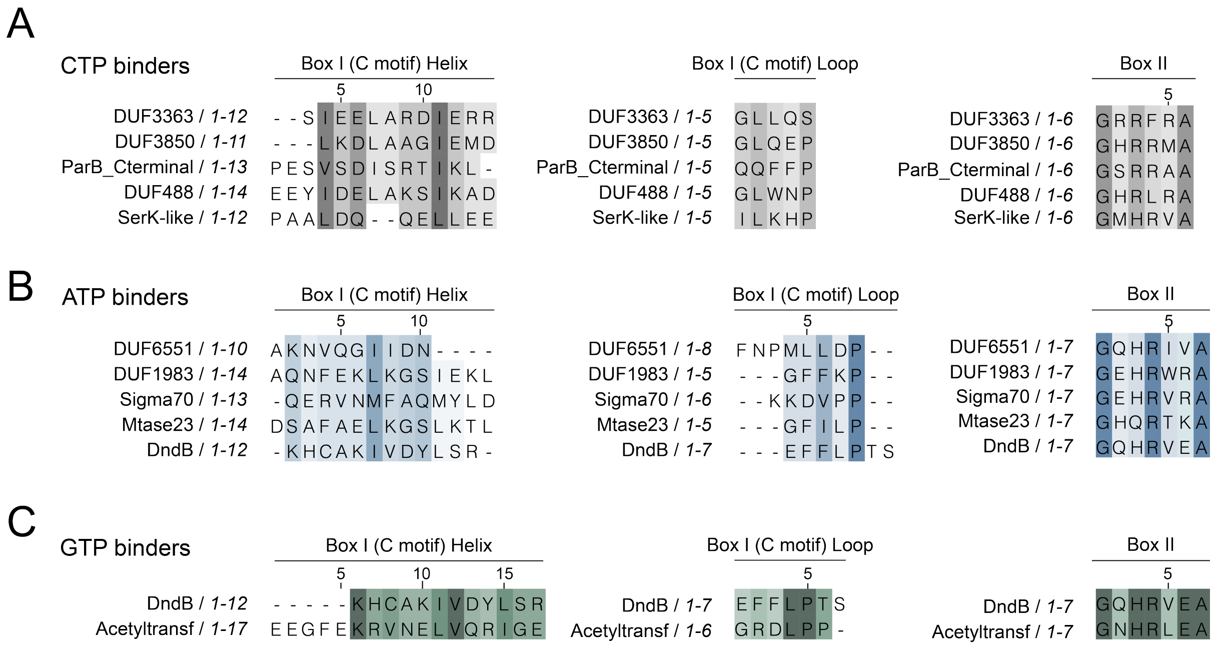

Fig. S6. Sequence alignments of conserved motifs in ATP-, CTP-, and GTP-binding ParB-like proteins. Related to Fig. 6. For each nucleotide-binding protein, AlphaFold2 structural models were used to identify Box I Helix, Box I Loop (C motif), and Box II (P motif). The sequences of these motifs were then extracted and aligned separately for CTP-, ATP-, and GTP-binding proteins. Alignments were performed using MUSCLE and visualized in Jalview, with residues coloured by conservation. *(A)* CTP binders (grey). Representative proteins include ParB-CTPase fold fused with DUF3363, DUF3850, ParB_C-terminal, DUF488, and SerK-like domains. *(B)* ATP binders (blue). Representative proteins include ParB-CTPase fold fused with DUF6551, DUF1983, Sigma70, Mtase23, and DndB domains. *(C)* GTP binders (green). Representative proteins include ParB-CTPase fold fused with DndB and Acetyltransferase domains. These alignments highlight subtle sequence features across binding classes and were used in preparation for Fig. 6*A-B*. See also Dataset S6.

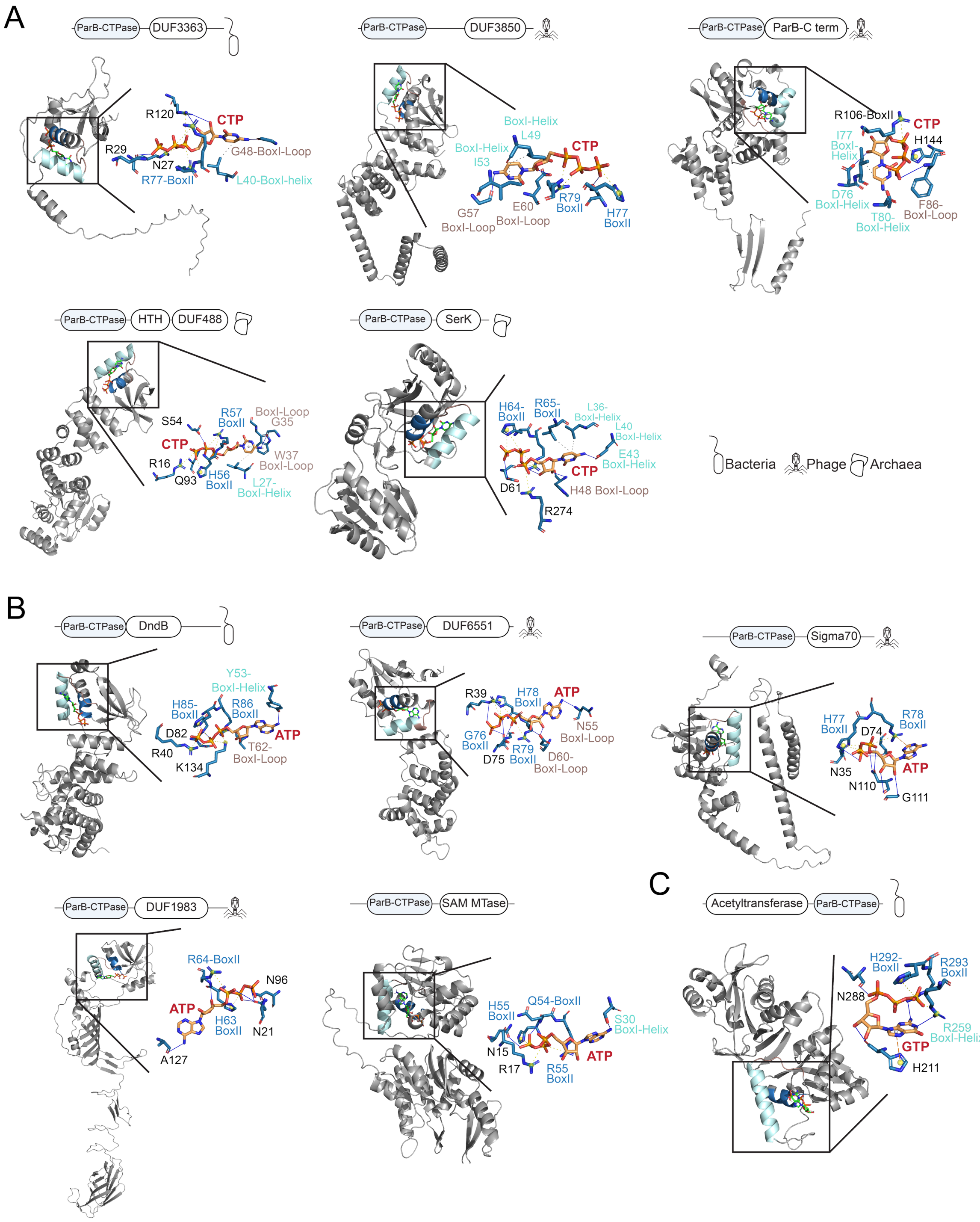
A
ParB-CTPase
DUF3363
R120
CTP
R29
N27
G48-BoxI-Loop
R77-BoxII
L40-BoxI-helix
DUF3850
BoxI-Helix
L49
BoxI-Helix
I53
CTP
G57
BoxI-Loop
E60
BoxI-Loop
R79
BoxII
H77
BoxII
ParB-C term
R106-BoxII
CTP
H144
I77
BoxI-Helix
D76
BoxI-Helix
T80-
BoxI-Helix
F86-
BoxI-Loop
HTH
DUF488
S54
R57
BoxII
BoxI-Loop
G35
CTP
R16
W37
BoxI-Loop
Q93
H56
BoxII
L27-
BoxI-Helix
SerK
H64-
BoxII
R65-
BoxII
L36-
BoxI-Helix
L40
BoxI-Helix
E43
BoxI-Helix
CTP
D61
H48 BoxI-Loop
R274
Bacteria
Phage
Archaea
B
DndB
Y53-
BoxI-Helix
H85-
BoxII
R86
BoxII
D82
ATP
R40
T62-
BoxI-Loop
K134
DUF6551
R39
H78
BoxII
ATP
N55
BoxI-Loop
G76
BoxII
R79
BoxII
D75
D60-
BoxI-Loop
Sigma70
H77
BoxII
D74
R78
BoxII
N35
ATP
N110
G111
DUF1983
R64-BoxII
N96
ATP
H63
BoxII
N21
A127
SAM MTase
H55
BoxII
Q54-BoxII
S30
BoxI-Helix
N15
R17
R55
BoxII
ATP
C
Acetyltransferase
H292-
BoxII
R293
BoxII
N288
R259
BoxI-Helix
GTP
H211

Fig. S7. Related to Fig. 6. Structural predictions and docking of ATP-, CTP-, and GTP-binding ParB-CTPase fold-containing proteins. *(A-C)* AlphaFold3 models of the 11 ParB-CTPase fold proteins that showed positive nucleotide binding in DRaCALA were used for molecular docking with their respective ligands. Proteins are grouped by binding preference: *(A)* CTP binders, *(B)* ATP binders, *(C)* GTP binders. For each protein, the schematics on the top show domain organization, with the ParB-CTPase fold in blue and fused domains in white. Icons indicate the source of the protein (bacteria, phage, or archaea). In the structural models, the ParB-CTPase fold is shown with Box I Helix (C motif) in cyan, Box I Loop (C motif) in maroon, and Box II (P motif) in blue. Insets show zoomed docking poses of the ligand (CTP, ATP, or GTP, in red) and predicted interacting residues. Residues are labelled according to whether they originate from Box I Helix and Box I Loop (C motif), or Box II (P motif). See also Dataset S6.

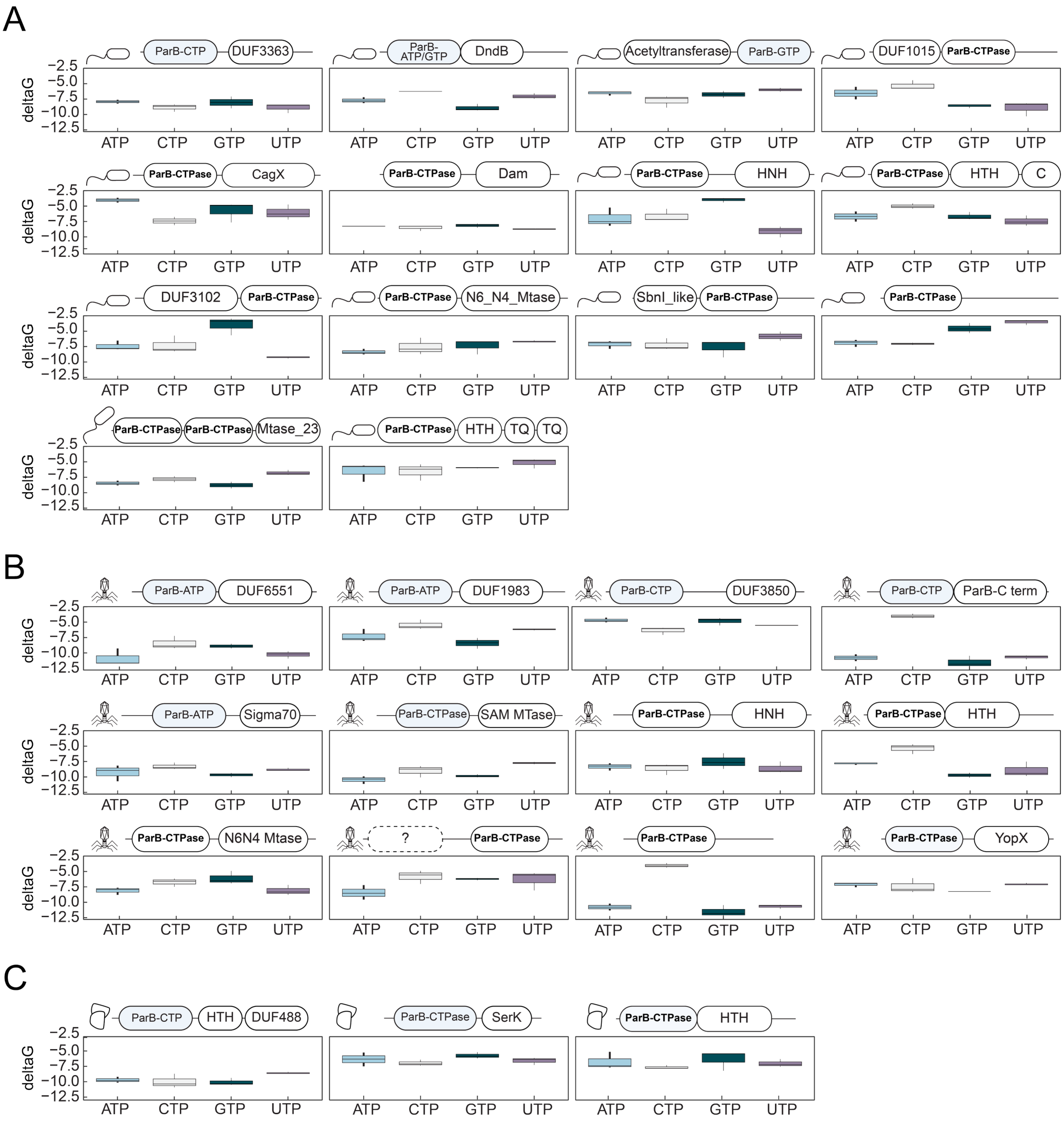

Fig. S8. AlphaFold3-predicted docking energies of nucleotides to ParB-CTPase fold-containing proteins. Related to Fig. 6. Predicted binding energies (ΔG, kcal/mol) for ATP, CTP, GTP, and UTP were obtained using AlphaFold3 docking for all proteins tested in DRaCALA assays (positive and negative binders; see Fig. 5 and *SI Appendix,* Fig. S5). Domain architectures are shown on top of the corresponding docking scores. For positive binders, the ParB-CTPase fold is highlighted in blue, and the experimentally confirmed ligand is written. For negative binders, the ParB-CTPase fold is shown in white. Proteins are grouped by origin: bacteria *(A)*, archaea *(B)*, and phages *(C)*.

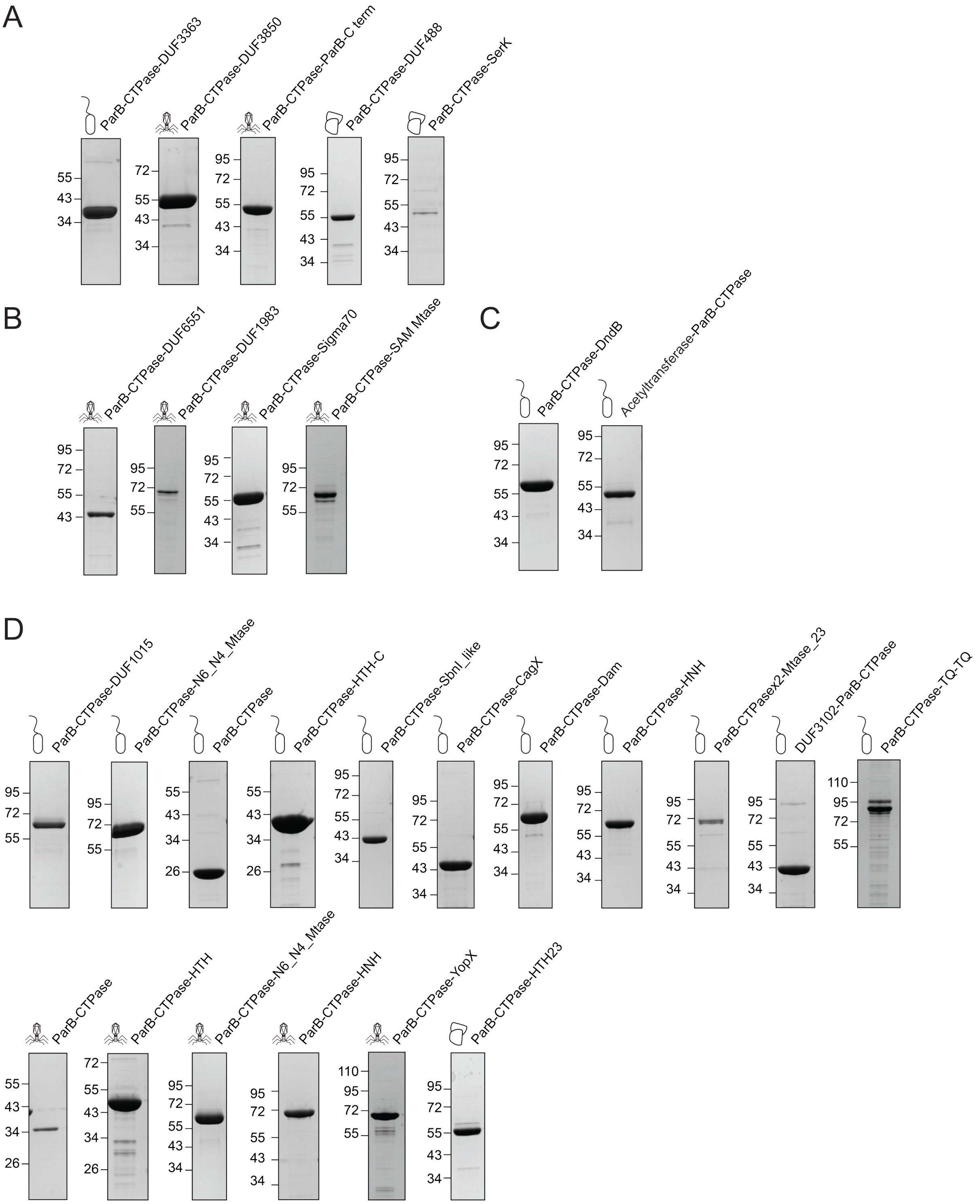

Fig. S9. SDS-PAGE analysis of purified ParB-CTPase proteins tested for NTP binding. (*A-C)* Representative SDS–PAGE gels showing comparable purification yields of the proteins tested in Fig. 5. *(A)* CTP-, *(B)* ATP-, and *(C)* GTP-binding proteins. *(D)* Proteins showing no detectable NTP binding, corresponding to *SI Appendix*, Fig. S5. All proteins were expressed in *E. coli* and purified under identical conditions. Comparable band intensities across positive and negative binders indicate that differences in NTP binding are not due to variation in protein expression or purification efficiency.
